# Ten simple rules for being a faculty advocate of first-year graduate students

**DOI:** 10.1371/journal.pcbi.1009379

**Published:** 2021-09-30

**Authors:** Kevin A. Janes

**Affiliations:** 1 Department of Biomedical Engineering, University of Virginia, Charlottesville, Virginia, United States of America; 2 Department of Biochemistry & Molecular Genetics, University of Virginia, Charlottesville, Virginia, United States of America; Carnegie Mellon University, UNITED STATES

## Introduction

The first year of graduate school is hard—new location (usually), new peers, new expectations [1]. It can be especially difficult for students who are starting their PhD with health challenges, student debt [2], imposter syndrome [3], or a background outside the “Research 1” (R1) Carnegie Classification [4]. If there are impediments to making the undergraduate-to-graduate transition, or there is no prior context to draw from, is it really all that surprising to see some students struggle? PhD advisors should help pave the way, but they usually do not become deeply involved until the end of a coursework-heavy first year. More direct engagement with faculty could help but runs up against the issue that there are only so many hours in the day. Any change would need to be highly circumscribed for it to be durable.

Toward improving the first-year experience of all graduate students, my colleagues and I developed the Faculty Advocates Program in the Department of Biomedical Engineering at the University of Virginia. The Program pairs an incoming student with a faculty member who will advocate for and help shepherd the student through their first year. The interactions are geared to set the student on a path to becoming their own best advocate for the rest of graduate school. The Faculty Advocate cannot be their advisor, but it should be someone with a broad interest in graduate student success and in promoting diversity, equity, and inclusion within the field. The streamlined format consists of six precisely timed one-on-one meetings during the academic year (Table 1). The meetings are confidential unless legal reporting requirements are triggered (e.g., Title IX). For those interested, further details are available about the scope and preliminary outcomes of the Faculty Advocates Program on a diverse cohort of first-year graduate students (S1 Text). In principle, it is applicable to any science, technology, engineering, and mathematics (STEM) graduate program where the number of willing faculty participants can map to the number of interested first-year students.

**Table 1:** A Faculty Advocates Program comprised of six one-on-one meetings.

Meeting	Timing	Purpose
1	Mid-August	Arrival on campus and start of Fall term
2	Early October	Debrief first laboratory rotation
3	Early November	Debrief second laboratory rotation
4	Early December	Debrief third laboratory rotation; selection of thesis advisor
5	Late March	Check in midway through Spring term
6	Early May	Debrief Spring term and discuss upcoming summer

When we debuted the Program in 2019, there was considerable interest from faculty but also hesitancy about what Faculty Advocates should be doing with their paired student. The two might not have the same scientific interests or career aspirations—how could they know if they were being helpful? Inspired by the “Ten Simple Rules” series of *PLOS Computational Biology*, I drafted this set of guidelines for colleagues piloting the Faculty Advocate Program. The feedback was positive, and among the Ten Simple Rules involving mentorship or diversity [5–8], there was nothing quite like it; so, time to share. The list came about from my years as a “plastic” advisor of PhD students [9], a member of several challenging thesis committees, and the extended mentor of a neuroscience student through the University of Virginia Mentoring Institute, which seeks to foster the success of students from traditionally underrepresented backgrounds. The Rules are not laws but rather considerations for when one embarks on a short-term, proactive relationship with a brand-new graduate student. The list is ordered chronologically as a sequence of things to think about along the way. Many extend to later-stage students seeking additional support beyond their PhD advisor. Of course, the Rules are subject to change as I continue learning how to improve as a Faculty Advocate.

## Rule 1: Assume nothing

I frequently tell students in class that the only dumb question is the one that does not get asked. The same holds in a new student–advocate pairing. It is good to establish a rapport of open inquiry with your student early on. Asking—rather than assuming—helps the advocate avoid substituting their expectations or prior knowledge in place of their student’s. I prefer questions that demand meaningful answers. For example, a student’s response to, “What is your single biggest concern for the first year of graduate school?”, will always be more illuminating than their answer to something like, “How are classes going?” In addition, it is good to inquire about whether the student is facing any health, financial, or personal burdens that could impact their first year. The question should be framed tactfully to avoid forcing the issue while making clear that no burden should be considered off-limits. That way, the door is open for the student to disclose what they choose. For many students, such openness feeds back rapidly to the advocate. Typically, a little encouragement is all that is needed to alleviate insecurity about the first year. Upon hearing the advocate ask such basic questions without hesitation, the student is more comfortable to reciprocate with honest questions of their own.

## Rule 2: Talk less, listen more

Like most faculty, I would talk to the wall if I thought it would listen. In six student–advocate meetings (Table 1), if I am doing most of the talking, then I have turned my student into a wall. Given a chatty student, Rule 2 is easy to follow, but, often, the students who have the most to gain from advocacy are the ones least likely to speak. Avoid the inclination to conclude that such students are uninterested or disengaged in the whole activity. Usually, they are just shy or come from a culture where respect means being deferential to one’s elders. To get the ball rolling, think about some open-ended questions such as those described in Rule 1. Regardless of upbringing, I have found that most young people do not like silence during a meeting—faculty can use this to their advantage in goading a student to speak! To promote self-advocacy, be deliberate about positively reinforcing them to voice their ideas and their perspectives. Negative reinforcement, especially early on, will cause the student to shut down and poison the developing relationship.

## Rule 3: Success is subjective

Another favorite question of mine is, “What is your definition of success in graduate school?” I have never heard the exact same answer twice. Some look to outputs, others to outcomes, and still others to the training experience itself. The upshot is that it is impossible to predict, so don’t try to guess—ask. If success metrics are misaligned between student and faculty, then it can create a big hurdle to achieving a sustainable goal of self-advocacy. A Faculty Advocate wastes two people’s time by suggesting or implying that graduate school success is toward R1 tenure-track positions or bust. Conversely, they can be a great help in laying out the professional expectations and milestones needed to reach a student’s success target. The point is to have students succeed on their terms, not yours. Remember whose career we are talking about.

## Rule 4: Empathize to connect

Upon reading Rules 1 to 3, a faculty member may come to expect that they will have nothing in common with their new student. Not true! No matter how long ago, you were a student once too. The uncertainty, confusion, frustration, or disappointments in a student’s first year will likely have some parallels to your own if you search for them and recount freely. Advocates who speak openly about their own vulnerabilities are much more relatable when discussing how they put themselves forward in graduate school. Students often feel like they are “on an island,” and it is powerful to hear about how others were once in their place. Plus, everyone likes a good story. Don’t contort yourself if common ground is simply not there; also, recognize that a faculty’s perception of an old, resolved challenge will be different from a student’s perception of a current one. Nevertheless, offering empathy gives students the option to receive it, and even the simple affirmation of an issue can have a strong positive impact on the student’s approach to it.

## Rule 5: Don’t give answers; help them be found

As principal investigators, we routinely adopt the role of problem-solver: why did this experiment not work, how do we address that reviewer critique, what is the most important next step for a project, etc. Achieving self-advocacy would be more straightforward if the same approach could be applied, but it cannot. Mathematically, problem-solving = dependency ≠ self-advocacy. Instead of a problem-solver, think as a guide whose role is to lay out the possible paths, talk about what lies ahead of each of them, and explain how the student is empowered to make the choice. These discussions are not quick fixes toward maximizing productivity but foundational conversations that lay the groundwork for long-term success. The ironic goal of a Faculty Advocate is to eventually render themselves unnecessary. That cannot happen if one is a perpetual fixer.

## Rule 6: You are a voice, not the voice

In the eyes of most students, their Faculty Advocate is an influential person. So are their lab rotation advisors, their colleagues, their family, and their friends. Together, those different perspectives can be a choir or a cacophony. Faculty should be aware of the notes and undertones that others are contributing to their student; it will affect their trajectory to becoming a self-advocate. This is particularly important for the student that listens too well—the one who tries to follow everyone’s advice instead of reconciling different perspectives before making a coherent decision (see equation in Rule 5). Knowing about these other voices will enable the advocate to craft an orchestrated piece of advice rather than a solitary one. In the end, the goal of self-advocacy is to find one’s own voice.

## Rule 7: Translate

Combining Rules 2 and 6 yields Rule 7. Faculty Advocates do a great early service to their students when they deconstruct, explain, or rephrase the actions, inquiries, or advice of other faculty. Nine times out of ten, you know the perspective of that faculty better than the student. Maybe the other faculty is pushing for a grant deadline or is stressed about teaching a new class for the first time. Maybe their advising style is more hands-off, hands-on, or hands-over compared to what the student has experienced before. Maybe the student just needs to hear the same advice delivered in a slightly different manner. Regardless of the circumstances, the role of translator is a valuable one to fill. A student who learns to be a truly effective listener will find it easier to be heard down the road. Beyond the faculty, there are also cultural differences within a department that could be very different from what the new student has experienced before. A Faculty Advocate can succinctly explain the norms that promote a sense of belonging and describe strategies the student can consider if they would like to distinguish themselves.

## Rule 8: Look forward more than backwards

Occasionally, it is worthwhile for a Faculty Advocate to revisit the past in search of lessons learned, but time with a student should mostly be spent on the near- and intermediate-term future. Students are penalized when they miss out on an opportunity simply because they were not prepared to take advantage of it. Showing effective planning by demonstrating it in front of your student is a powerful catalyst for self-advocacy. For instance, after learning about their success metrics (Rule 3), I set out a realistic target date for that success. Then, I work backwards with them to illustrate how success in Years 4 and 5 requires specific efforts in Years 1 and 2. Doing so reinforces how long-term outcomes are determined by a series of short-term activities and assertions. It also keeps the Faculty Advocate proactive about helping their student avoid problems instead of fixing the ones that have already arisen (Rule 5).

## Rule 9: Care

A common refrain in parenting is that it doesn’t matter to your child if you burn the toast or put a diaper on backwards, as long as you are clearly trying your best to care for them. There are some parallels with the Faculty Advocates Program. Students in distress feel that they have been abandoned. They perceive success in everyone around them and fear being left behind; the feeling is especially acute if it occurs in that first year of graduate school. To have a senior figure believe in them, recognizing all the challenges they face, is an enormous catalyst for them to persevere and grow. How you demonstrate that belief and that caring toward your student is entirely up to you—there is almost no wrong way to do it. A goal for Faculty Advocates is to do it early and proactively enough so that students avoid distress altogether (Rule 8).

## Rule 10: Be direct

Rule 10 could have been Rule 1. It is possible to be direct (Rule 10), to be kind (Rule 9), and to be humble (Rule 6). For example, once I have a sense of a student’s intrinsic strengths and weaknesses, I tell them both in equal measure and ask if they agree. If a student surpasses a developmental milestone or is stalling at one, I tell them as soon as I see it and explain why I believe it is important for their definition of success (Rule 3). Among all, Rule 10 can be the hardest to follow for faculty who dislike having uncomfortable conversations. If a first-year student has an unrealistic vision of graduate school success, would you say so or let it slide to avoid stifling them? Get over it—your student’s best interests are at stake (Rule 9). They need to know exactly what you think.

## Conclusions

Being a Faculty Advocate to first-year graduate students is a dual-use activity. In the same way that teaching can make us better scientists, short and consistent interactions with brand-new students can make us better advisors and communicators if we make it a priority—all the more so if these students come from backgrounds traditionally underrepresented in science and engineering. The challenge for a Faculty Advocate is that time is short and there are so many roles to fill: guide (Rule 5), conductor (Rule 6), translator (Rule 7), augur (Rule 8), and quasi-parent (Rules 2, 4, 9, and 10). It is impossible to do perfectly, and some student–advocate relationships simply do not work out (S1 Text). However, when it does, it can have a lasting impact on both student and faculty for the better.

The Faculty Advocates Program and these Ten Simple Rules have limitations. I write from a privileged R1 institution about a graduate program where our trainees are adequately stipended. In my field, there are many career opportunities outside of the academic track that we endorse as faculty. My remarks thus paint too rosy a picture of graduate student support across all institutions and all graduate programs. From that broader standpoint, there is a strong argument that faculty guidance for graduate students should remain focused on money and jobs (see the peer review history of this Editorial). The Ten Simple Rules described here do not generalize to faculty–student interactions in all STEM disciplines and cannot salvage relationships that have become dysfunctional. What I hope they can provide is a starting point for considering how faculty can engage with first-year graduate students differently and sustainably.

## Supporting information

S1 Text Supporting information about participation and survey outcomes from the Faculty Advocates Program in the Department of Biomedical Engineering at the University of Virginia. (DOCX)
