# Supplementary material for: Ten simple rules for being a faculty advocate of first-year graduate students
Source: PLoS Comput Biol. 2021 Sep 30;17(9):e1009379. doi: 10.1371/journal.pcbi.1009379 (PMC8483399; doi:10.1371/journal.pcbi.1009379)
Supplement: S1 Text — (DOCX) [file pcbi.1009379.s001.docx]

**SUPPORTING INFORMATION**

**Ten Simple Rules for Being a Faculty Advocate of First-Year Graduate Students**

Kevin A. Janes^1,2^*

^1^ Department of Biomedical Engineering, University of Virginia, Charlottesville, Virginia USA

^2^ Department of Biochemistry & Molecular Genetics, University of Virginia, Charlottesville, Virginia USA

* Email: [kjanes@virginia.edu](mailto:kjanes@virginia.edu)

**S1 Text. Details about the Faculty Advocates Program implemented in the Department of Biomedical Engineering at the University of Virginia**

Formally, our one-year program should be called a Faculty (Mentors for Self-)Advocates Program. The faculty-student interactions are timed so that the student can hit the ground running when they arrive on campus and make the most of their laboratory rotations during the first semester. A common discussion topic is how a student can stand up for themselves and their own self-interests when interacting with a rotation supervisor or Ph.D. advisor. Many first-year students, and supervisors alike, are unclear about expectations for a rotation—Faculty Advocates explain what is otherwise left between the lines (Rule 7). The cycle for fellowships and awards runs from the Fall (NSF Graduate Research Fellowships) to the Spring (NIH Training Grant Fellowships)—Faculty Advocates anticipate the timelines so that students are poised to compete (Rule 8). For us, the best first-year advocacy is to be proactive in charting out the early steps to success so that students can see the course ahead of them.

| Year | Graduate students | | Faculty | |
| --- | --- | --- | --- | --- |
|  | Number | % of total | Number | % of total |
| 2019 | 16 | 44% | 15 | 54% |
| 2020 | 26 | 65% | 19 | 68% |
| 2021 | 21 | 55% | 21 | 75% |

**S1 Table.** Participation statistics for the Faculty Advocates Program in the Department of Biomedical Engineering at the University of Virginia. Graduate students = all M.E., M.S., and Ph.D. students in the Department. Faculty = all primary appointments in the Department.

Thus far, we have assembled three cohorts for the Program and collected survey data on two. Buy-in for the Program has been considerable: ~55% of entering graduate students typically opt-in to participate (15–25% of whom come from underrepresented backgrounds), and the percentage of faculty engagement has increased each year (S1 Table). Student-advocates pairings are arranged roughly by research field, but several advocates are so adaptable that they can be paired effectively with any first-year student. The overall risk of any student-advocate pairing is limited by the one-year scope of the Program; at worst, the effect of a suboptimal pairing should be neutral.

**S1 Fig.** Improved perceptions of the Faculty Advocates Program when one-on-one meetings are held at least four times in one year. **(A)** Stratification of student-advocate responses. **(B)** Perceived utility of the one-on-one meetings from students and faculty. **(C)** Likelihood of recommending the Faculty Advocates Program to future colleagues. Differences were assessed by Fisher’s exact test.

Response rates to the exit survey (64% overall) were substantive enough to analyze the 2019–2020 data in aggregate, combining faculty and student responses for both years. Roughly half of the respondents reported meeting at least four times during the year, providing a balanced stratification (<4 vs. ≥4) to assess whether meeting frequency corresponds to overall satisfaction with the Program. Student-advocate pairings that met more frequently were more likely to feel that their total number of meetings was appropriate (S1A Fig). Even with as many as six meetings, no respondent indicated that the commitment was “Too much”. Interestingly, the ≥4 group also showed a significantly stronger indication that their meetings were highly productive and useful (S1B Fig). The impact of student-advocate meetings thus does not appear to get diluted out by a somewhat larger number of meetings. We observed a similar effect on how strongly participants would recommend the program to others in the future (S1C Fig). These observational associations cannot disambiguate whether pairings that met more frequently were more likely to be successful or whether intrinsically successful pairings were more likely to meet frequently. Nevertheless, the results indicate a threshold effect that will inform future offerings of the Faculty Advocates Program at the University of Virginia.
